# Supplementary figures and images for: Identification and safety assessment of Enterococcus thailandicus TC1 isolated from healthy pigs
Source: PLoS One. 2021 Jul 1;16(7):e0254081. doi: 10.1371/journal.pone.0254081 (PMC8248690; doi:10.1371/journal.pone.0254081)

**S1 Fig.**


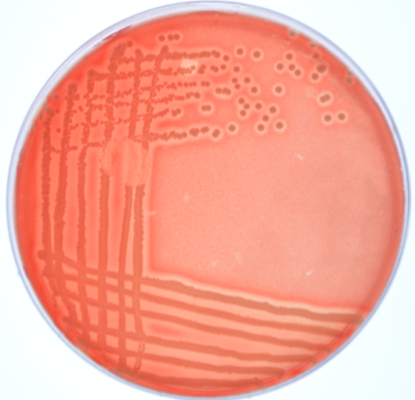

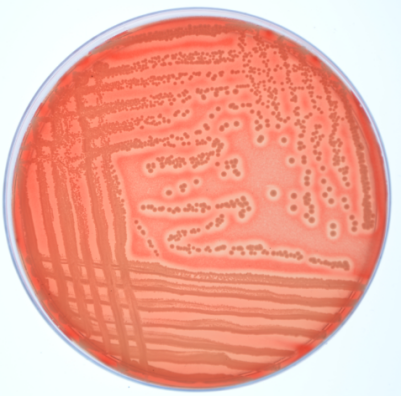

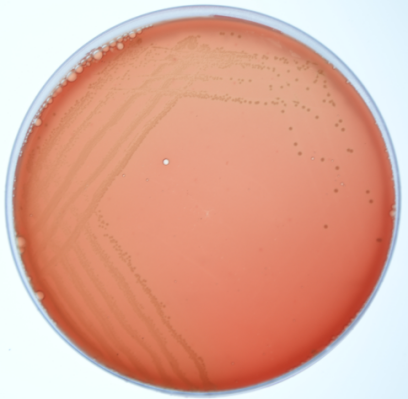

Supplement: S1 Fig — 1: Positive control; 2: The isolated strain; 3: Negative control. (DOCX) [file pone.0254081.s001.docx]
