# Supplementary material for: Identification and safety assessment of Enterococcus thailandicus TC1 isolated from healthy pigs
Source: PLoS One. 2021 Jul 1;16(7):e0254081. doi: 10.1371/journal.pone.0254081 (PMC8248690; doi:10.1371/journal.pone.0254081)
Supplement: Attachment — Submitted filename: Response to Reviewers.docx [file pone.0254081.s003.docx]

Dear Editor Rosa del Campo,

Thank you very much for your decision letter and advice on our manuscript (Manuscript # PONE-D-21-10442R1) entitled “Identification and safety assessment of *Enterococcus* *thailandicus* TC1 isolated from healthy pigs”. We also thank the reviewers for the constructive comments and suggestions. We have revised the manuscript accordingly, and all amendments are indicated by red font in the revised manuscript. In addition, our point-by-point responses to the comments are listed below this letter.

We hope that our revised manuscript is now acceptable for publication in your journal and look forward to hearing from you soon.

With best wishes,

Yours sincerely,

Xiliang Wang

**Replies to Editor**

1. Writing the names of the bacteria is as important as being rigorous in the experiments. After the reviewers and myself have pointed out the errors, major mistakes still remain. The first time a microorganism is named, the name should be in full: Enterococcus thailandicus, but from this point on it should be contracted to E. thailandicus, of course in italics.

**Response**: Thank you for your suggestion. The mistakes were modified in revised manuscript as marked in red color.

2. Other errors are in the word "pstD" in line 31 which is in another style of letter, a space before the bracket in line 62, line 79 aureus should be in italics, and the final points of the statements of the tables.

**Response**: Thank you for your suggestion. The mistakes were modified in revised manuscript as marked in red color.
